# Enhancing Path Planning Capabilities of Automated Guided Vehicles in Dynamic Environments: Multi-Objective PSO and Dynamic-Window Approach

**DOI:** 10.3390/biomimetics9010035

**Published:** 2024-01-05

**Authors:** Thi-Kien Dao, Truong-Giang Ngo, Jeng-Shyang Pan, Thi-Thanh-Tan Nguyen, Trong-The Nguyen

**Affiliations:** 1 Fujian Provincial Key Laboratory of Big Data Mining and Applications, Fujian University of Technology, Fuzhou 350118, China; 1101405123@nkust.edu.tw; 2 School of Computer Science and Mathematics, Fujian University of Technology, Fuzhou 350118, China; 3 Multimedia Communications Laboratory, University of Information Technology, Ho Chi Minh City 700000, Vietnam; 4 Vietnam National University, Ho Chi Minh City 700000, Vietnam; 5 Faculty of Computer Science and Engineering, Thuyloi University, Hanoi 116705, Vietnam; 6 College of Computer Science and Engineering, Shandong University of Science and Technology, Qingdao 266510, China; jspan@cc.kuas.edu.tw; 7 Faculty of Information Technology, Electric Power University, Hanoi 100000, Vietnam; tanntt@epu.edu.vn

**Keywords:** automated guided vehicles, path planning, multi-object PSO, dynamic-window approach, collision avoidance, energy consumption, travel time

## Abstract

Automated guided vehicles (AGVs) are vital for optimizing the transport of material in modern industry. AGVs have been widely used in production, logistics, transportation, and commerce, enhancing productivity, lowering labor costs, improving energy efficiency, and ensuring safety. However, path planning for AGVs in complex and dynamic environments remains challenging due to the computation of obstacle avoidance and efficient transport. This study proposes a novel approach that combines multi-objective particle swarm optimization (MOPSO) and the dynamic-window approach (DWA) to enhance AGV path planning. Optimal AGV trajectories considering energy consumption, travel time, and collision avoidance were used to model the multi-objective functions for dealing with the outcome-feasible optimal solution. Empirical findings and results demonstrate the approach’s effectiveness and efficiency, highlighting its potential for improving AGV navigation in real-world scenarios.

## 1. Introduction

Automated guided vehicles (AGVs) have emerged as pivotal assets across diverse industries, particularly in settings (e.g., warehouses and manufacturing facilities) where their prowess in efficiently transporting goods within controlled environments is highly valued [1]. AGVs operate by adhering to predefined routes or employing navigation algorithms to delineate their trajectories [2]. However, in intricate and dynamic surroundings, path planning for AGVs becomes a formidable undertaking [3], necessitating the simultaneous optimization of multiple objectives, notably encompassing energy conservation, travel duration, and collision avoidance [4].

Path planning for AGVs has been the subject of extensive inquiry within the academic literature [5]. Various algorithms [6] including A* search [7], Dijkstra’s algorithm [8], and rapidly-exploring random trees (RRT) [9] have been harnessed to ascertain the optimal routes for AGVs. Nevertheless, these conventional methodologies may fall short in addressing the dynamic nature of environments and the multifaceted objectives associated with AGV operations.

The contemporary state-of-the-art in AGV path planning embodies a multifaceted landscape that integrates cutting-edge technologies and methodologies [10]. One notable trajectory in AGV path planning encompasses combining traditional algorithms with modern machine learning techniques. Combining established algorithms like A* search [11] with reinforcement learning (RL) [12], hybrid approaches enable AGVs to navigate static and dynamic terrains with remarkable adaptability. Deep reinforcement learning (DRL) [13], in particular, has garnered considerable attention, permitting AGVs to learn and optimize paths through iterative interactions with their environment [14].

Meta-heuristic algorithms are a class of optimization algorithms inspired by natural phenomena such as genetic algorithms [15,16], differential evolution (DE) [17,18], swarm intelligence [19,20] (ant [21] and bee [22] colonies, wolf optimization [23]), and simulated annealing (SA) [24] that have been applied to various optimization problems including path planning for AGVs. Meta-heuristic algorithms can handle high-dimensional and nonlinear optimization problems with multiple objectives and constraints. Meta-heuristic algorithms can also provide robust and adaptive solutions that can deal with environmental uncertainties and disturbances.

Table 1 compares the advantages and disadvantages of contemporary state-of-the-art AGV path planning.

Furthermore, integrating real-time sensor data has become a hallmark of advanced AGV path planning [37]. Equipped with sensors such as LiDAR [38], cameras, and ultrasonics [39], AGVs have acquired an enhanced perception of their surroundings. This heightened situational awareness empowers AGVs to implement precise collision avoidance strategies and dynamic path planning. Machine learning algorithms, trained on sensor data, contribute to interpreting and predicting environmental changes, further reinforcing the AGV navigation capabilities in complex scenarios [40].

The primary quandary in AGV path planning resides in achieving equilibrium amid the optimization of numerous objectives while circumventing collisions and flexibly adapting to ever-changing environments [41]. This necessitates identifying an approach that efficaciously navigates the intricacies of multi-objective optimization [42] and seamlessly integrates real-time environmental information [43].

The aims of this study are listed as follows: to develop a path planning system for AGVs that successfully optimizes various goals including energy use, travel time, and collision avoidance. Incorporating real-time environmental data into their trajectory planning improves the flexibility of AGVs in dynamic contexts [44] and enhances the effectiveness and efficiency of AGV path planning in complex and dynamic environments. This study introduces a pioneering approach amalgamating multi-objective particle swarm optimization (MOPSO) and the dynamic-window approach (DWA) [45] for AGV path planning. The MOPSO algorithm facilitates the optimization of multiple objectives [46], while the DWA technique [47] imparts real-time adaptability to changing environmental conditions. The salient contributions of this paper encompass the following.

- The integration of MOPSO and DWA enables the efficient management of multi-objective optimization challenges and dynamic environmental dynamics in AGV path planning.
- Empirical assessments of the proposed approach across diverse scenarios, substantiating its effectiveness in optimizing objectives, averting collisions, and accommodating environmental fluctuations.
- Comparative analyses against extant methodologies to demonstrate the superiority of the proposed method in terms of efficiency, effectiveness, and adaptability.

The proposed approach promises to significantly enhance the AGV path planning capabilities significantly, fostering heightened operational efficiency and safety within industrial domains.

The rest of this paper’s structure unfolds as follows. Section 2 delves into related work, providing comprehensive reviews of AGVs, particle swarm optimization (PSO), and the dynamic-window approach (DWA). Section 3 expounds upon the methodology employed in this research. Section 4 outlines the experimental setup and presents the results and discussion. Finally, Section 5 encapsulates the conclusions drawn from this study.

## 2. Related Work

This section reviews the relevant literature regarding automated guided vehicles (AGVs) and presents the particle swarm optimization (PSO).

### 2.1. Automated Guided Vehicles

Automated guided vehicles (AGVs) are autonomous vehicles that transport goods within industrial facilities [1,48]. In recent years, significant advancements have been made in AGVs, revolutionizing how goods are transported in various industries [11]. Researchers have focused on improving the efficiency, safety, and adaptability of AGVs to meet the evolving demands of modern production systems, logistics, and transportation. Integrating cutting-edge sensing technology like LiDAR [38] and computer vision is one noteworthy advancement that has made it possible for AGVs to perceive and navigate complicated situations with greater accuracy and dependability.

Previous work has opened the door for more effective path-planning algorithms that optimize AGV trajectories while considering various factors including energy consumption, travel duration, and collision avoidance. AGVs are equipped with sensors such as laser scanners and vision systems to perceive their environment and navigate safely. AGVs have gained popularity due to their ability to improve efficiency, reduce labor costs, and enhance safety in material handling operations. Path planning techniques for AGVs have been proposed for AGVs that can be broadly categorized into traditional and intelligent optimization algorithms [48]. Standard algorithms include A* search, Dijkstra’s algorithm, and RRT, which rely on predefined maps and graph-based planning methods. Intelligent optimization algorithms, on the other hand, employ optimization techniques to find optimal paths based on various objectives [49].

**Figure 1:**
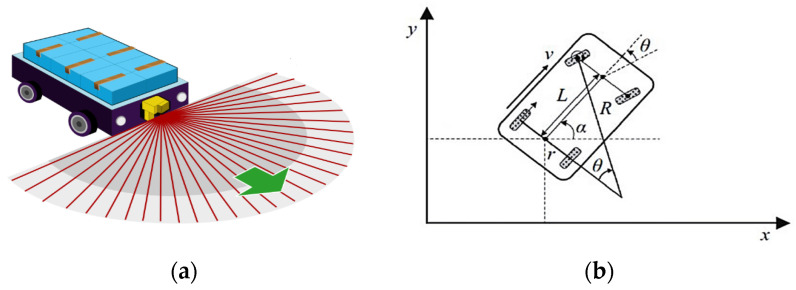
Illustration of a typical automated guided vehicle (AGV) and the wheel angles in arc path planning tracking. (**a**) Automated Guided Vehicle (AGV); (**b**) AGV driving wheels angles in tracking arc path.

Figure 1 illustrates a typical automated guided vehicle (AGV) and wheel angles in arc path planning tracking, where (a) shows the AGV and wheel angle calculation in CRC path planning.

The AGV is responsible for transporting items within a given space, moving from the starting point to the desired destination [50]. To ensure efficient and obstacle-free movement, the AGV must be able to navigate around static obstacles while maintaining the shortest possible path and minimizing the steering angles. This requirement is necessary to meet multiple objectives simultaneously. The AGV workspace is defined as a physical area represented by RxR, where obstacle mapping is undertaken of the workspace section that is free from collisions and allows for movement between the AGV and obstacle. It is assumed that the AGV has access to environmental information about its workspace [48]. In Figure 1b, let *α* and *θ* be the angle between the vehicle body and the *X*-axis, respectively, the steering wheel angle with the assumed two wheels are parallel and satisfy the Ackermann steering geometry, and *L* is the wheelbase [1]. A moving AGV distance in the static obstacle at adjusted speed represents the shortest path between points, which follows the given formula.

(1) dist=min∑i=1n(xi+1−xi)2+(yi+1−yi)2, s.t. a=vtanθL, x=vsinα−ycosα, y=xsinθ+α−ycosθ+α−Lacosθ,x=vsinαy=vcosα 

where *α* and *θ* are the vehicle body and the *X*-axis angle and the steering wheel angles, respectively; horizontal *x* and vertical *y* present the velocity components of the axis, respectively; dist is a distance path between points; n is the number of steering angles in total.

The direction of motion of the AGV vehicle body is influenced by various physical factors such as gravitational acceleration, running velocity, and vehicle mass [51]. Additionally, in a dynamic environment of obstacles in the working space, the speed of the AGV is limited by factors like curvature, turning radius, maximum steering speed, static friction coefficient, and minimum radius. The AGV steering is subject to the following procedure with the motion constraints. Therefore, the AGV running velocity is subject to the following formula with an acceleration with a limiting minimum radius of the speed.

(2) vmax=u·g·1+L2.k2R2−L2, s.t. ξmin≥m.vmax2R, vi≤vmax, k=1R ,  

where $v_{i}$ and $v_{max}$ are the running velocity and maximize running velocity, respectively; u and g are static friction coefficient and gravitational acceleration parameters, respectively; $v_{i}$ and m are the running velocity and vehicle mass variables, respectively; R and k are the deployed area measured length, curvature turning radius, and maximum steering speed variables, respectively.

### 2.2. Particle Swarm Optimization (PSO)

A population-based optimization system called particle swarm optimization (PSO) was inspired by the cooperative behavior of fish schools and bird flocks [19]. In the PSO, a collection of particles stands in for potential solutions, and these particles search a search space for the best possible answers: a particle’s individual best-known solution and the swarm’s overall most prominent solution impact how it moves [52]. There are several phases of the PSO running process—initialize a swarm population, update the particle’s position and velocity, evaluate the objective function of the particle’s new position, update personal and global bests, and check termination conditions are some examples of the PSO process optimization.

The ***initialization phase*** is implemented to initialize a swarm of particles with random locations or positions and velocities within the search space. In this phase, the personal best position is also set, and the fitness of the objective function for each particle to its initial position and fitness.

(3) $$\left\{\begin{array}{c} S=unifrnd\left(Ub,Lb,d\right),\;\\ V=zeros\left(d\right),\;\\ Fitness=CostFunction\left(S\right),\; \end{array}\right.$$

where S and V are initialized for the location as the position and velocity of the PSO particles, respectively; Fitness is evaluated as the fitness value for particle position based on cost function or objective function; Ub and Lb are the problem area searching space boundaries; unifrnd is a random generating function; d is dimension. The particle with the best fitness is identified as the global best position and fitness.

The ***updating phase*** for the particle’s velocity and position is carried out with each particle in the whole population.

(4) $$V_{id}^{\left(t+1\right)}=\omega .V_{id}^{t}+\alpha .\lambda_{1}\left({pbest}_{id}^{t}-S_{id}^{t}\right)+\beta .\lambda_{2}\left({gbest}_{d}^{t}-S_{id}^{t}\right),$$

where $\lambda_{1}$ and $\lambda_{2}$ are the generating randoms in arrangement [0, 1]; ω presents the inertia weight; d is the dimension; ${pbest}_{id}^{t}$ and ${gbest}_{d}^{t}$ are variable vectors for presenting the personal and global best positions of the particle’s with the current generation, respectively; α and β present the acceleration coefficients. Particle positions S are updated using the following formula.

(5) $$S_{id}^{\left(t+1\right)}=V_{id}^{t}+S_{id}^{t},$$

where $S_{id}^{t}\;$ and $V_{id}^{\left(t+1\right)}$ present the particles’ position and velocity at t, which is the current generation or iteration and index of particle id-the.

The evaluation phase: Using the fitness function unique to the problem being solved, the assessment phase is carried out by assessing the fitness of each particle’s new position. After that, the personal and global bests can be updated, for instance, by comparing the fitness of each particle to its personal best fitness and updating the personal best position and fitness if the current fitness is higher. If necessary, the particle’s fitness is compared to the global best fitness, updating the global best position and fitness. The termination condition phase determines whether the termination condition has been satisfied such as reaching the required fitness level or the maximum number of iterations. The algorithm is stopped if the termination condition is satisfied; else, return to the *updating phase*.

Algorithm 1 illustrates a particle swarm algorithm optimization as its pseudo-code PSO.
**Algorithm 1:** A pseudo-code PSOInput: particles *V*, *S*, *Np*, and fitness function Output: Solution as global best position *S*1. 2. 3. 4. 5. 6. 7. 8. 9. 10.Start Initializing particles: Equation (3) Initializing global best position and fitness
While termination condition is not met   For each particle     Updating particle’s velocity: Equation (4)     Updating particle’s position: Equation (5)     Evaluating fitness of the particle’s new position     Updating personal best position and fitness     Updating global best position and fitness     End

Moreover, there have been various improvements in PSO versions, and several related strategies in multi-objective optimization represent different strategies for enhancing the performance of MOPSO in solving multi-objective optimization problems. These aim to improve the search space exploration, prevent premature convergence, and adapt the algorithm’s parameters to the situation. Each approach has advantages and may be more suitable for different optimization problems. The parallel MOPSO approach [53] involves running multiple instances of MOPSO simultaneously on different processors or cores to solve a single optimization problem. Each instance operates independently, combining their results to find an overall solution. This approach can significantly reduce the computational time required for optimization. Multi-swarm PSO [54] uses the particles’ population divided into multiple subgroups or swarms, each with its own leader. The swarms interact and exchange information to explore the search space more effectively and prevent premature convergence to suboptimal solutions.

Adaptive PSO involves dynamically adjusting the parameters of the MOPSO algorithm based on the performance of the particles during the optimization process [55]. This can help improve the convergence speed and the quality of the solutions. In MOPSO, with the improved strategy of selecting the global particle guides used, the selection of international guides for the particles is improved to enhance the exploration and exploitation capabilities of the algorithm, which can lead to better convergence and more accurate solutions.

## 3. Results of MOPSO-DWA for Enhancing AGV Path Planning

This section presents a suggested integration of MOPSO and DWA for enhancing AGV path planning via several subsections (e.g., reviewing the dynamic-window approach (DWA), stating multi-objective PSO with modeling objective function, and combining the schemes of integration MOPSO and DWA).

### 3.1. Dynamic-Window Approach (DWA)

**Figure 2:**
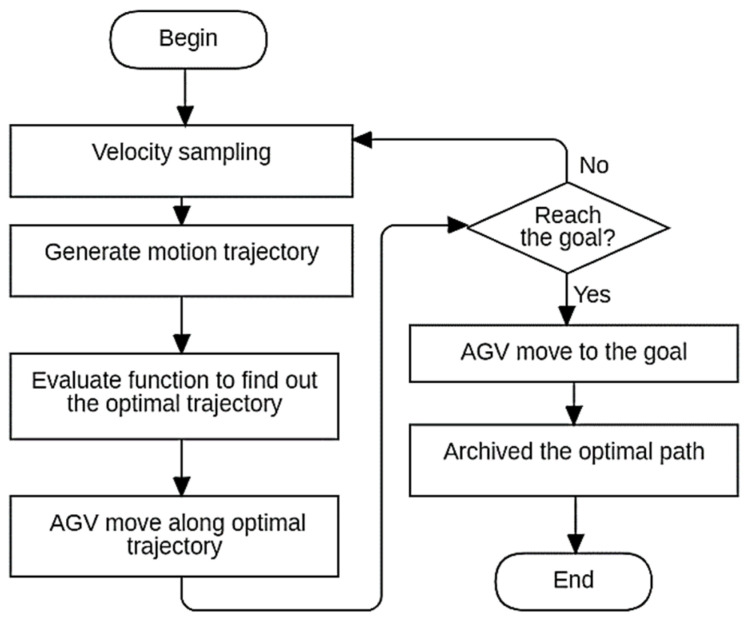
A flowchart for the local path planning scheme’s DWA technique.

The dynamic-window approach (DWA) is the reactive navigation method within the proposed path-planning approach [47] that allows the AGV to reactively navigate in real-time, considering the current state of the environment and the AGV’s capabilities [56]. It involves the following steps. (1) Generate a dynamic window of allowable velocities based on the AGV’s maximum and minimum velocities. (2) Sample a set of potential velocities from the dynamic window. (3) For each velocity sample, simulate the AGV’s trajectory over a short time horizon. (4) Calculate a score for each trajectory based on predefined criteria such as collision avoidance, proximity to the goal, and smoothness of motion. (5) Select the highest-scoring trajectory as the following action for the AGV. (6) Execute the selected action and update the AGV’s state. Step (7) is repeatedly undertaking the following action. The DWA is a reactive navigation method developed for mobile robots [57]. It works by sampling a set of potential velocities and computing their corresponding trajectories to assess their quality based on predefined criteria such as collision avoidance and proximity to the goal. The highest-scoring trajectory within a dynamically changing window is selected as the following action. DWA allows robots including AGVs to reactively navigate in real-time, considering the current state of the environment and the robot’s abilities. Figure 2 shows a flowchart of the DWA method of the local path-planning algorithm.

Here are the detailed steps of the DWA algorithm:

*Step 1*—Sensing and localization: Obtain sensor readings to perceive the environment and estimate the robot’s current position and orientation.

*Step 2*—Motion prediction: Predict the future motion of the robot based on its current state and dynamics model. Consider the robot’s velocity, acceleration, and steering capabilities.

*Step 3*—Dynamic-window generation: Define a dynamic window representing the robot’s feasible velocity and steering combinations. Set the maximum and minimum limits for linear and angular velocities based on the robot’s capabilities.

*Step 4*—Trajectory evaluation: Generate a set of candidate trajectories by sampling velocities within the dynamic window. Evaluate each trajectory based on different criteria such as proximity to obstacles, proximity to the goal, and smoothness of motion. Assign a score or cost to each course based on the evaluation criteria.

*Step 5*—Trajectory selection: Select the trajectory with the lowest cost or highest score as the desired trajectory for the robot. Ensure that the selected trajectory is collision-free and satisfies the dynamic constraints of the robot.

*Step 6*—Motion execution: Convert the desired trajectory into control commands for the robot’s actuators. Execute the control commands to move the robot along the desired trajectory.

*Step 7*—Loop: Continuously repeat Steps 1 to 6 to update the robot’s motion plan based on the changing environment and robot state. Adjust the dynamic window and re-evaluate trajectories to adapt to the dynamic nature of the domain.

The DWA algorithm enables the robot to navigate safely and efficiently in dynamic environments by iteratively generating and evaluating trajectories within a dynamic window. It accounts for the robot’s dynamic constraints, obstacle avoidance, and goal-reaching objectives to generate appropriate motion plans.

### 3.2. Multi-Object Particle Swarm Optimization (MOPSO)

The optimal AGV path planning is applied via the MOPSO in the dynamic-window approach technique. In managing multiple objectives of the MOPSO, a swarm of particles with different positions and velocities indicates potential solutions [46]. The particles traverse the search space for the best answers that optimize the specified objectives [42]. The following phases comprise the MOPSO algorithm [46]: initialization randomly initializes the particle locations and velocities, evaluation for the goals established for AGV path planning, assessment of each particle’s fitness, update personal best and global best based on the individual best locations of each particle, update the global best position using the individual and collective best agent solutions, update the velocities and positions of the particles, and whether the termination criterion is satisfied.

Multi-objectives consist of two objective functions: the shortest path objective function and the maximum smoothness objective function. The shortest path objective function is modeled by calculating the distances along identified points to guide the AGV forward or backward, turning or moving, as path planning. The first objective function of the shortest path is expressed as follows.

(6) $$F_{1}\left(n\left(ta\right),P_{i}\left(x_{i},y_{i}\right),P_{i+1}\left(x_{i+1},y_{i+1}\right)\right)=min\sum_{i=1}^{n(ta)}\sqrt{{\left(x_{i+1}-x_{i}\right)}^{2}+{\left(y_{i+1}-y_{i}\right)}^{2}},$$

where $F_{1}$ is the objective function of the shortest path; $P_{i}$ is the location in the working area space of *i*-the point; n(ta) is the total number of steering angles of moving AGV; $x_{i}$ and $y_{i}$ are the axis of the *i*-the point of velocity of the AGV horizontal and vertical present.

The second objective function is the aim of the maximum smoothness function, which is presented as follows.

(7) $$\begin{array}{l} \;F_{2}\left(n\left(ta\right),P_{i}\left(x_{i},y_{i}\right),P_{i+1}\left(x_{i+1},y_{i+1}\right)\right)=\\ min\sum_{i=1}^{n(ta)}{{(P}_{i-1}\left(x_{i-1},y_{i-1}\right),P}_{i}\left(x_{i},y_{i}\right),P_{i+1}\left(x_{i+1},y_{i+1}\right)).\left|\arctan\left(\frac{y_{i+1}-y_{1}}{x_{i+1}-x_{i}}\right)-\arctan\left(\frac{y_{i}-y_{i-1}}{x_{i}-x_{i-1}}\right)\right|, \end{array}$$

where $F_{2}$ is the objective function of the shortest path; $x_{i}$ and $y_{i}$ are the axis of *i*-the point of velocity of the AGV horizontal and vertical with the arctan calculation for $P_{i}$ and $P_{i+1}$ in the working area space of the moving AGV. As arctan angles are used as the basis for steering, they are thus determined by the path’s smoothness, which has a similar meaning to curvature. Shorter pathways and more minor alterations result from increased smoothness correlated with reduced curvature.

Several methods can establish multi-objectives, for example, by using the weighting parameter and Pareto optimal solution.

The weighting parameter with multi-objective function is expressed as follows.

(8) $$F=\omega \times F_{1}+(1-\omega )\times F_{2},$$

where F is a multi-objective function with $F_{1}\;$ and $F_{2}$; ω is a parameter of weighting for balancing objectives.

Multi-objective optimization relies on the concept of Pareto print optimization to optimize objectives simultaneously. Pareto dominance is a crucial concept in multi-objective optimization, allowing for the simultaneous optimization of multiple goals. This involves analyzing various aspects of the multi-objective model such as Pareto disaggregation or optimal solutions, the solution set, and non-inferior solutions. A Pareto dominance comparison is performed among the particles to determine dominance relationships. Additionally, each particle in each front is assigned a crowding distance to maintain diversity. Finally, dominance ranks are assigned to the particles based on their Pareto dominance relationships.

Method multi-objective optimization is carried out by adapting multi-objective PSO optimization (MOPSO), which is expressed in detailed steps as follows.

*Step 1*—Initialization: The positions and velocities of a swarm of particles are randomly generated within the search space. The personal best position and fitness of each particle of the initialized position are set as the initial values. The particle identifies the global best position with its best fitness.

*Step 2*—Updating the particle’s velocity and position: The V velocity of each particle is updated by using Equation (4). Solution S as the position is updated by the particle’s position using Equation (5).

*Step 3*—Evaluation: The objective function of or fitness of each particle’s new position is calculated by multiple objective functions: *F*1—Equation (6) and *F*2—Equation (7), each representing a different objective for evaluation.

*Step 4*—Updating the personal and global bests: This is carried out by comparing the fitness value with its personal best fitness for each particle objective. If the current fitness is better for any objective, update that objective function’s personal best position and fitness value. Furthermore, computation of the global best fitness for each objective is carried out by comparing the fitness value of the particle with whether the current fitness is better for any objective function and updating the global best position and fitness for that objective.

*Step 5*—Pareto dominance: Pareto dominance is performed by comparing the particles to determine the dominance relationships. Assign dominance ranks to the particles based on their Pareto dominance relationships. The weights are dynamically adjusted based on the particles’ performance and the optimization problem characteristics based on the distribution of solutions along the Pareto front.

*Step 6*—Non-dominated sorting: The particles into different non-dominated fronts are sorted based on their dominance ranks, expressed as the following formula.

(9) $$L_{i}=\sum_{n=1}^{N}\left(\frac{f_{n}^{i+1}-f_{n}^{i-1}}{f_{n}^{max}-f_{n}^{min}}\right),\;i=2,3,\ldots ,k-1,$$

where $f_{n}^{max}$ and $f_{n}^{min}$ are the max and min values in the Pareto solution set of earcg objective functions; N (here *N* is set to 2) is the total number of objective functions; *k* is the total number of non-inferior solutions. A crowding distance to each particle is assigned in each front to maintain diversity.

*Step 7*—Selection and reproduction: Particles from the non-dominated fronts are selected based on their dominance ranks and crowding distances. Reproduce new particles by combining the chosen particles through crossover and mutation operations.

*Step 8*—Termination condition: This is executed by checking if the termination condition is met (e.g., reaching a maximum number of iterations or reaching the desired target). If the termination condition is met, stop the algorithm; otherwise, go back to Step 2.

### 3.3. Integration of MOPSO and DWA

The proposed approach integrates the MOPSO algorithm and DWA, enabling efficient and effective path planning for AGVs. In combination with MOPSO and DWA for AGV path planning, MOPSO is used to optimize the selection of velocities and trajectories within the DWA framework, allowing for efficient and adequate decision-making in real-time—the significance of AGV path planning and the various techniques employed to optimize the selection of paths. Integrating MOPSO and DWA presents a promising approach to address the challenges of AGV path planning in complex and dynamic environments.

The integration involves the following steps. Initialization: The MOPSO algorithm with a swarm of particles is initialized in the desired area space, each representing a potential solution for the AGV’s path. A dynamic window of velocities is generated based on the AGV’s capabilities. For each particle in the swarm, the following steps are performed: (a) Use the MOPSO algorithm to optimize the selection of velocities within the dynamic window based on multiple objectives; (b) apply the DWA approach to select the highest-scoring trajectory for the AGV based on the optimized velocities.

**Figure 3:**
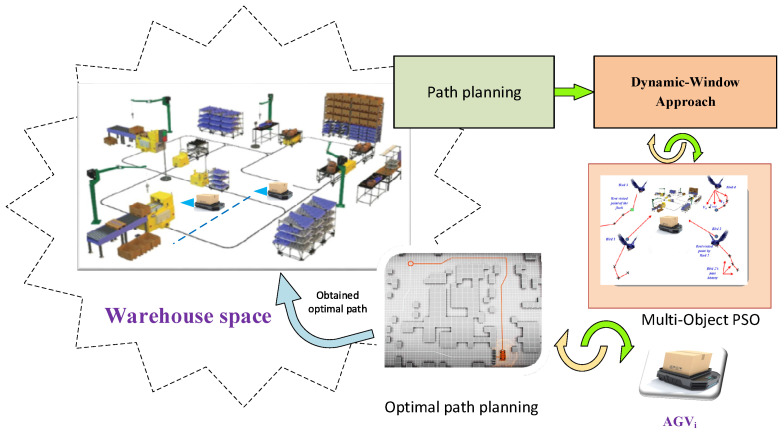
An overview of a MOPSO-DWA for enhancing an AGV’s path planning capabilities in a dynamic environment.

The next step is to update the positions and velocities of the particles using the MOPSO algorithm. Repeat generating new solutions and checking the fitness until the termination criteria are met (e.g., a maximum number of iterations or convergence). The final step is to select the best solution from the swarm as the optimal path for the AGV. Figure 3 illustrates an overview of MOPSO-DWA for enhancing an AGV’s path-planning capabilities in a dynamic environment.

**Figure 4:**
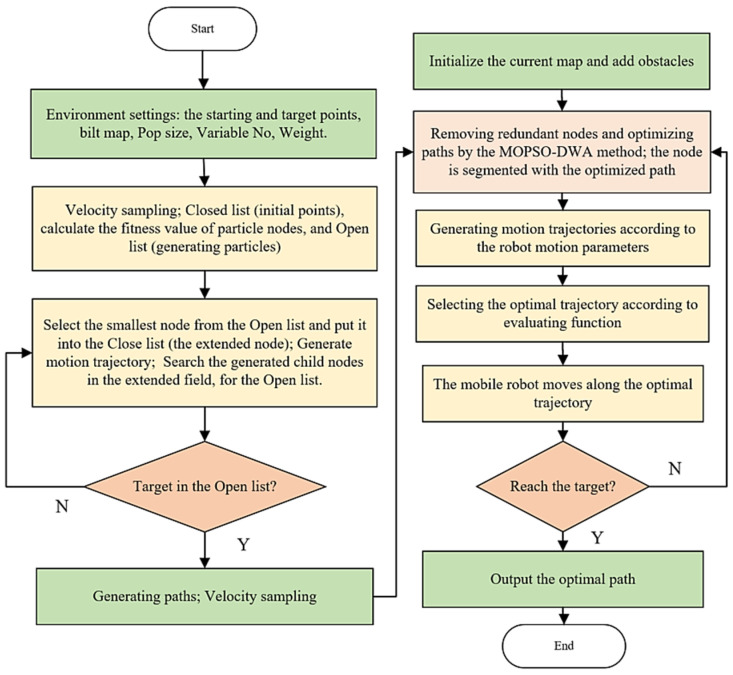
A flowchart of the suggested scheme for optimal path planning. Green denotes the generating input/output or initializing variables, yellow rectangles refer to processing procedures, and orange refers to conditions or emphasized proposals.

By integrating MOPSO and DWA, the proposed approach can optimize multiple objectives and dynamically adapt to changing environments, leading to efficient and safe path planning for AGVs in complex industrial environments. Figure 4 illustrates a flowchart of the suggested MOPSO-DWA scheme for optimal path planning.

The proposed path-planning approach for AGVs was applied to a design system architecture that comprised several components (e.g., sensor module, map and localization module, path planning module, control system, and AGV navigating platform). The suggested architecture includes (1) the sensor system: sensors (e.g., laser scanners and vision systems) can be used to perceive the environment and obtain real-time information; (2) the map and localization module (e.g., a created map of the environment) and the AGV’s position and orientation are determined using localization techniques; (3) the path planning module, where the proposed optimization is applied (e.g., the module generates optimal paths based on multiple objectives and real-time information); (4) the control system (e.g., the control module) converts the planned paths into control commands for the AGV’s actuators; (5) the AGV platform execute the control commands to navigate through the environment.

## 4. Experimental Results and Discussion

This section presents the simulation and experimental results through the subsections (e.g., the environmental work settings, performance metrics of the experimental results) as well as a discussion.

### 4.1. System Environmental Settings

System environmental settings were used to test the proposed approach’s performance at the same environment setting for a fair comparison. The results of the proposed approach were compared with existing path-planning approaches for AGVs (e.g., A star algorithm, genetic algorithm (GA) [26], particle swarm optimization (PSO) [19,28], multi-evolutionary algorithm (SPEA-strength Pareto evolutionary algorithm) [41], and multi-objective genetic algorithm (NSGA-II-Non-dominated sorting GA) [42,58]). The parameters were set for the multi-objective optimization methods (e.g., *Np*, the population size, was set to 100, the max_generations of iterations was 1000, the random learning factors were set to ω, *c*_1_, *c*_2_ for the PSO parameter settings, crossover, and mutation probability paraments were set to 0.8 and 0.2 for the genetic algorithm and evolutionary algorithm respectively, and the max steering velocity was set to 0.2 m/s. Two dimensions of *MxL* (*M* and *L* are unit length metrics for length and width) environment raster maps of different complexity were built in the environment, where the black part represents the known obstacles, the gray part represents the access node area, and the white part represents the passable area. Table 2 displays the brief configuration parameters used in the environmental experiment.

An involved system architecture of the proposed path-planning approach for AGVs is described with several component modules [3] and is a high-level overview of the suggested system architecture in the proposed AGV path-planning approach.

(1) Sensor module: This component includes various sensors such as LiDAR [38], cameras, and proximity sensors to perceive the environment and gather information about obstacles, paths, and other relevant data.
(2) Localization module: Responsible for estimating and updating the AGV’s position and orientation in real-time. Utilizes techniques like odometry, GPS, or simultaneous localization and mapping to provide accurate localization information.
(3) Mapping module: Generates and maintains a map of the environment that includes information like obstacles, paths, and other relevant features. It uses sensor and localization module data to create and update the map.
(4) Path planning module: Determines the optimal path for the AGV to navigate from its current position to the desired destination. It considers factors such as obstacle avoidance, path smoothness, shortest distance, or time constraints by utilizing the MOPSO algorithm.
(5) Trajectory planning module: The optimal planned path into a smooth and feasible trajectory for the AGV to follow the obtained path. It considers the AGV’s dynamic constraints such as acceleration, deceleration, and maximum velocity to generate a safe and achievable trajectory.
(6) Control module: The trajectory planning module receives the trajectory and generates control signals to actuate the AGV. Controls the AGV’s actuators such as motors or steering mechanisms to execute the planned trajectory. Monitors and adjusts the AGV’s motion based on sensor feedback and the environment.
(7) Communication module: Facilitates communication and coordination between the AGV and other entities such as a central control system or other AGVs. Enables exchanging information, commands, and status updates to ensure synchronized operations and efficient coordination.
(8) Central control system: A centralized control and coordination mechanism is provided for multiple AGVs in a fleet. Monitors and manages the overall operation of the AGVs, assigns tasks, and optimizes resource allocation. Integrates with the communication module to exchange information with individual AGVs.

The system architecture describes the components involved in the proposed AGV path-planning approach.

### 4.2. Experimental Results

In comparison with existing approaches in this subsection, the results of the proposed approach were compared with existing path-planning methods for AGVs (e.g., A* [11], GA [26], PSO [28], SPEA [41], and NSGA-II [58] methods). Both single-objective and multi-objective optimization methods aim to demonstrate the superiority of the proposed approach in terms of efficiency, optimality, and adaptability to complex industrial environments. The comparison is conducted based on performance metrics such as convergence, path length, execution time, collision rate, smoothness of motion, and goal-reaching rate.

**Figure 5:**
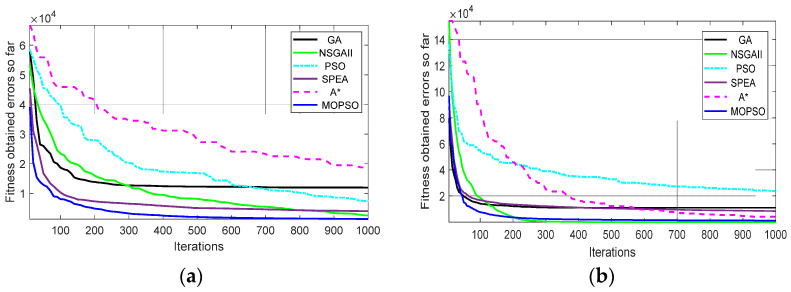
Comparison of the curve convergence rate of the proposed approach compared with the existing methods of the GA, PSO [28], SPEA, A* [11], and NSGA-II [58] algorithms for single-objective function. (**a**) Single objective function *F*1 (shortest path); (**b**) Single objective function *F*2 (smoothness path).

Figure 5 shows the curve convergence rate of the proposed approach compared with existing methods (e.g., A* [11], GA [26], PSO [28], SPEA [41], and NSGA-II [58]). The metric of errors was used in the scenario with the single objective function to evaluate the performance of the convergence test. It can be seen that the proposed approach of MOPSO had a convergence rate in both the separated single objective functions that were the smallest, which means that the proposed approach provides a fast converging speed.

**Figure 6:**
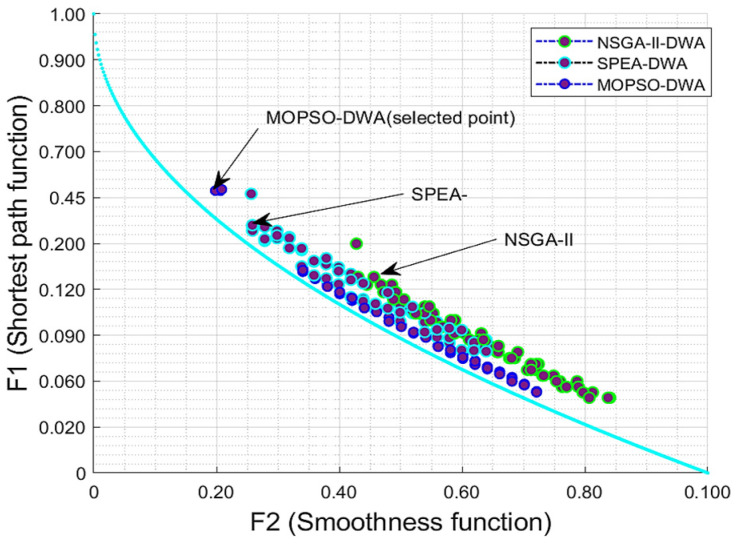
The Pareto optimal front with the obtained result curves of the multi-objective optimal AGV path planning from the MOPSO, SPEA, and NSGA-II algorithms. The blue line is the Pareto optimal front solutions.

The evaluation of multi-objective optimization was tested with the multi-objective optimization of MOPSO-DWA, which was made possible in AGV path planning by incorporating the MOPSO algorithm and DWA. The examination looked at the Pareto front produced by the MOPSO optimization over functions *F*1 (Equation (6)) and *F*2 (Equation (7)). It also illustrates the trade-off between goals such as the path length and motion smoothness as Equation (8). Figure 6 displays the Pareto optimal front with the obtained result curves of the multi-objective optimal AGV path planning from the MOPSO, SPEA, and NSGA-II algorithms. Observing Figure 6, most of the acquired optimal result points were allocated closer to optimizing the line of the Pareto optimal front than the other algorithms.

Further experiments were conducted in different scenarios, and the proposed approach’s performance could be thoroughly evaluated and compared with other existing path-planning methods, demonstrating its effectiveness and robustness in various industrial environments. Experimental scenarios can be used to evaluate the proposed approach comprehensively, and various experimental scenarios were created. The scenarios simulated different challenging situations and environmental conditions that AGVs may encounter in real-world industrial environments. Experiment scenario 1 compares the algorithm of the MOPSO-DWA approach with the environment with known static obstacles and the parameter value of the DWA algorithm, while experiment scenario 2 is to verify the obstacle avoidance performance of the MOPSO-DWA approach in an unknown obstacle environment.

Static obstacle avoidance is used to test scenarios with static obstacles placed in the environment to evaluate the AGV’s ability to avoid collisions and find the optimal paths around the obstacles. Dynamic obstacle avoidance tests scenarios where dynamic obstacles are introduced into the environment, representing moving objects or other AGVs. These test the AGV’s ability to dynamically react and adjust its path in real-time to avoid collisions.

**Figure 7:**
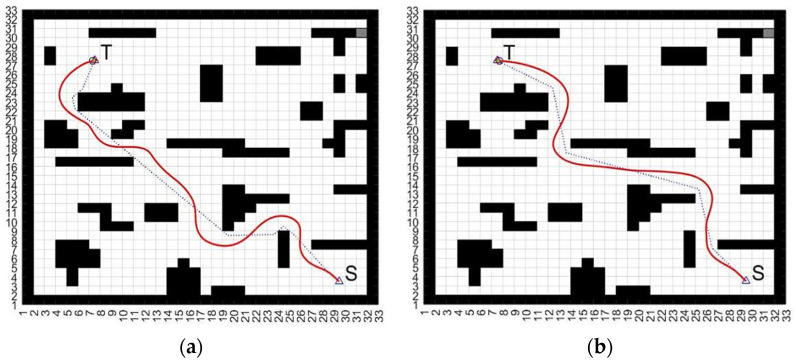
A comparison of the obtained path planning graph result of the proposed MOPSO-DWA approach with the A*-DWA method for static environment obstacle avoidance. S and T are the set start and target points, respectively. (**a**) A*-DWA approach; (**b**) MOPSO-DWA approach.

Figure 7 shows a comparison of the obtained path planning graph result of the proposed MOPSO-DWA approach with the A*-DWA method [11] for static environment obstacle avoidance.

The proposed approach of the integration of MOPSO-DWA for AGV path planning was evaluated using a simulation environment. The simulation environment provides a realistic representation of the industrial environment and allows for the testing and validating of the path-planning algorithms. The simulation environment included the following components: map, AGV, sensor, control simulations, and visualization. The map representation is prepared with an environment map that includes obstacles, goal locations, and other relevant features. The AGV simulation model is a model of the AGV that is implemented including its shape, size, kinematics, and dynamics. Sensor simulation is when the sensor system of the AGV such as laser scanners or cameras is simulated to provide real-time perception of the environment. Control simulation uses the control system of the AGV to execute the planned paths and generate control commands for the actuators. Visualization provides visual feedback on the AGV’s movements including its path, obstacles, and goal locations. Observing the obtained results from Figure 7, we can see that the MOPSO approach can provide the optimal global static route planning and successfully reach the goal point shorter than the A-star approach.

**Figure 8:**
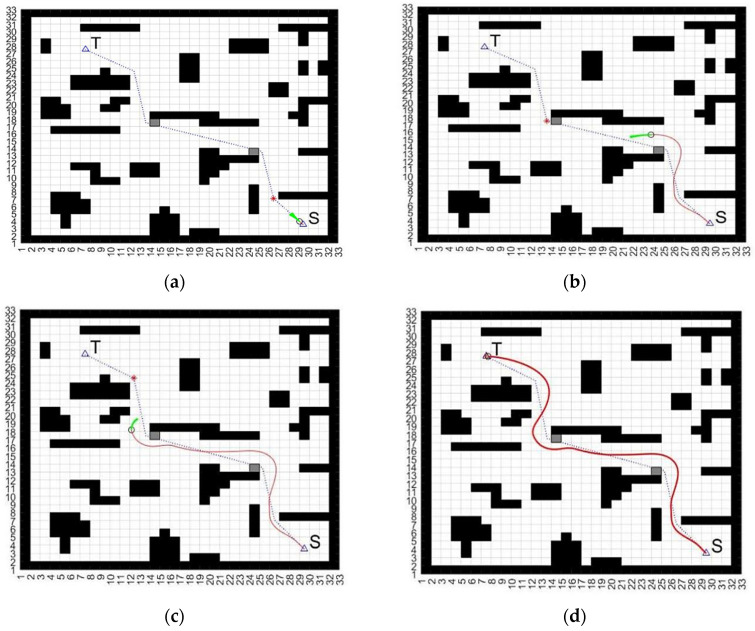
Processing applied AGV moving test in a dynamic environment, with unidentified obstacles represented as gray squares for real-time detection and avoidance. S and T are the set start and target points, respectively. (**a**) Obstacles adding (red point) to the path; (**b**) Avoiding the obstacle (previous red point); (**c**) Avoiding the second obstacle; (**d**) Reaching the target point.

In the other scenario of obstacles in a dynamic environment, the ability of the proposed approach to avoid obstacles in an environment with unknown obstacles was tested. The environment conditions were set to include obstacles in a dynamic environment, and anonymous blocks were added to the path to replicate these conditions. Figure 8 shows a test of the proposed approach in a dynamic environment, where the small gray squares represent these unidentified obstacles. This allows for real-time detection, avoidance, and effective routing.

Performance metrics can be applied to evaluate the proposed approach’s performance. Several metrics provide quantitative measures of the path-planning approach’s effectiveness and efficiency for AGV path planning (e.g., the path length, execution time, collision rate, smoothness of motion, and goal-reaching rate). A number of tests was conducted, repeated *N* times (*N* was set to 20 in the experiment). The results were calculated with the average of the obtained path length, execution time, collision rate, smoothness of motion, and goal-reaching rate. Table 3 compares the average results of several measures over *N* running times. The comparison with the existing path planning approaches for AGVs had the goal to demonstrate the superiority of the proposed approach in terms of the efficiency, optimality, and adaptability to complex industrial environments.

The values of the comparison of algorithms in Table 3 are displayed with statistics analysis calculations (e.g., path length (m), path planning time (s), path node, and path inflection point). The end of the table summarizes the effective rates. The comparison was conducted based on the performance metrics. For example, the path length is a metric of the length of the generated path from the start to the goal location. A shorter path indicates a more efficient and direct route.

First, it achieved a shorter path length than the NSGA and SPEA methods, positioning it as an efficient approach for optimizing the path length. It can be seen that the enhanced method also reduced the planning path length, which was reduced around 5.76% with other approaches, and for the goal-reaching rate metrics, it increased to 97%. Interestingly, it is on par with the A-star algorithm regarding the path length, indicating its competitive performance in this aspect.

Moreover, the proposed MOPSO method showcases the fastest path planning time among the compared methods, highlighting its efficiency in generating optimal paths in a timely manner. Additionally, it exhibited a superior collision rate and path node performance, outperforming the other methods in these critical metrics. The execution time is a metric of the time the AGV takes to navigate from the start to the goal location. A shorter execution time indicates faster navigation.

Furthermore, the MOPSO method excels in the goal-reaching speed and smoothness of motion, surpassing the other methods in these aspects. This suggests that the proposed MOPSO-DWA method optimizes the path length and planning time and enhances the overall quality of motion, leading to smoother and faster goal-reaching capabilities.

The collision rate is a metric of the percentage of collisions during path execution. A lower collision rate indicates safer navigation. The smoothness of motion measures the jerk or acceleration changes along the planned trajectory. The smoother motion means better control and comfort. The goal-reaching rate is the percentage of successful goal-reaching attempts. A higher goal-achieving rate indicates successful navigation. The proposed approach’s performance can be thoroughly evaluated and compared with other path-planning methods, demonstrating its effectiveness and robustness in various industrial environments.

### 4.3. Discussion Results

The impact of multi-objective optimization is manifested with the Pareto front obtained from the integration of MOPSO and DWA in AGV path planning, demonstrating the proposed approach’s superiority in terms of efficiency, optimality, and adaptability to complex industrial environments. The analysis examined the Pareto front obtained from the MOPSO optimization, as shown in Figure 6, by representing the trade-off between different objectives such as the path length and smoothness of motion. By analyzing the Pareto front, insights can be gained into the impact of different objective weights on the AGV’s path planning performance. The different objective weights affect the trade-off between the path length and smoothness of motion, providing insights into the optimal objective weights that lead to the desired balance between conflicting objectives. Integrating MOPSO with the dynamic-window approach allows for multi-objective optimization in AGV path planning, which helps determine the optimal objective weights that lead to the desired trade-off between conflicting objectives, enhancing the optimality of the proposed approach.

The effectiveness of the DWA can be evaluated by the effectiveness and efficiency of the DWA in selecting the highest-scoring trajectory for AGVs. The different window sizes impact resolutions on the built map in path planning performance. Additionally, the ability of the DWA to handle dynamic obstacles and adapt trajectory selection in real-time was examined. The evaluation helps determine the optimal parameters for the DWA, ensuring efficient and safe path planning for AGVs.

Performance metrics such as the path length, execution time, collision rate, smoothness of motion, and goal-reaching rate were analyzed to assess the effectiveness of the proposed approach. Each metric was examined individually to understand the improvements achieved by the proposed approach compared to existing approaches. The analysis highlights the significance of each metric in AGV path planning and how they contribute to the overall efficiency, optimality, and adaptability in complex industrial environments. The findings can be used to evaluate the proposed approach’s superiority over existing approaches in AGV path planning and emphasize the improvements achieved in terms of efficiency, optimality, and adaptability, supported by the analysis of performance metrics, multi-objective optimization, and the effectiveness of the DWA.

Limitations and future research directions for this study: Although the proposed approach shows promise, there are several areas for future research to enhance AGV path planning. One avenue is to explore advanced optimization algorithms such as GA, PSO, NSGAII, and SPEA to improve the multi-objective optimization performance. Additionally, accounting for environmental uncertainties such as sensor noise or incomplete information can lead to the development of robust path-planning algorithms. Furthermore, integrating machine learning techniques like reinforcement learning can enable AGVs to learn and adapt their path-planning strategies in response to changing environmental conditions. Finally, conducting real-world experiments and validations will provide practical insights into the implementation and performance of the proposed approach.

The proposed approach makes several contributions to the field of AGV path planning. First, the integration of multi-objective optimization enables AGVs to find the optimal paths by considering multiple objectives simultaneously. It provides more flexibility and adaptability in different industrial environments. Second, the dynamic-window approach effectively handles dynamic obstacles and adjusts trajectories in real-time, ensuring safe and efficient navigation.

## 5. Conclusions

This study introduced a pioneering approach for AGV path planning, integrating the multi-objective PSO (MOSPO) and dynamic-window approach (DWA) to overcome the limitations of traditional autonomous navigation methods for mobile AGVs. Our modification strategies facilitated the calculation of the optimal speed and steering angles, ensuring safe navigation. We effectively balanced conflicting objectives and improved the overall performance by mathematically modeling a multi-objective function by using the shortest path and maximum smoothness objective functions. Key factors such as the heuristic function, search direction, path safety, and redundant path nodes contributed to enhanced efficiency, optimized paths, and reduced inefficiencies. The dynamic-window approach successfully selected the highest-scoring trajectory, considering both the optimized velocities and environmental obstacles. Through rigorous simulations and comparisons with existing methods, we validated the effectiveness of our approach, demonstrating advantages such as a shorter planning time, reduced path length, fewer turning points, and increased route safety. Furthermore, our evaluation of the performance metrics provided quantitative measures of the approach’s effectiveness and efficiency, enabling objective comparisons with other AGV path-planning methods and addressing challenges related to efficiency, optimality, adaptability, and safety.

Looking ahead, our future research will focus on exploring advanced optimization algorithms to enhance multi-objective optimization performance, addressing environmental uncertainties such as sensor noise or incomplete information to develop robust path-planning algorithms, integrating machine learning techniques like reinforcement learning to enable AGVs to adapt their path-planning strategies to changing environmental conditions, and conducting real-world experiments and validations to gain practical insights into the implementation and performance of our proposed approach.

## Figures and Tables

**Table 1:** Contemporary state-of-the-art AGV path planning with their advantages and disadvantages.

Approach	Applications	Advantages	Disadvantages
A* algorithm [7,11]	Robotics, AGV navigation, path planning	Optimal path, widely used	Not suitable for dynamic environments
Dijkstra’s algorithm [8,25]	Robotics, AGV navigation, graph search	Optimal path, simple implementation	High computational complexity
Genetic algorithm [15,26,27]	AGV routing, optimization, logistics planning	Robust optimization, handling uncertainties	Slow convergence, parameter tuning required
Particle swarm optimization [19,28]	AGV path planning, optimization, multi-objective	Multi-objective optimization, adaptability	Lack of global search capability
Dynamic programming [29]	AGV navigation, optimal control, robotics	Optimal path, efficient computation	Limited scalability for large environments
Reinforcement learning [30]	AGV path planning, adaptive navigation, robotics	Adaptive path planning, learning from experience	High training time, potential for suboptimal paths
Differential evolution [17,18]	Optimal path planning for mobile robots	Single-objective optimization, adaptability	Not adaptable environments
Simulated Annealing [24,31]	A single optimization objective for optimal robot path planning	Use polylines, pline interpolated, and Bézier curves for modeling fitness function.	Inadaptable global search capability
Optimal path planning using hybrid PSO-SA [20]	Multi-objective optimization for optimal control, robotics	Factors of the minimized path length and smooth path for modeling fitness function	Insufficient extent for significant search capability
Ant colony optimization [32] and its variants [21,33]	Mobile shortest path with obstacle avoidance	Using adaptive fuzzy control, angle guidance factor	Limited enlargeable environments with scalability search development.
Combination of ABC [22] with Evolutionary programming for path planning [34]	AGV navigation, optimal control, robotics	Adaptive path planning, learning from experience	Execution time, potential for suboptimal paths
Grey wolf optimization [23,35,36]	Optimal mobile robot path planning	Single-objective optimization, adaptability model	Limited scalability, executing time, potential long

**Table 2:** Parameter settings used in the conducted environmental experiment.

Algorithm	Parameters Setting
GA [26]	Mutation rate $P_{m}$ = 0.1, crossover rate- $P_{c}$ = 0.8, *Np* = 100, tournament size τ = 5, max_gen = 1000
NSGA-II [42,58]	Crossover rate- $P_{c}$ = 0.85, *Np* = 100, tournament size τ = 5, max_gen = 1000, mutation rate $P_{m}$ = 0.1,
SPEA [41]	*Np* = 100, Archive size $A_{r}=100,$ max_gen = 1000, mutation rate $P_{m}$ = 0.1, crossover rate- $P_{c}$ = 0.85
PSO [19,28]	$V_{max}=10,V_{min}=-10,\omega \in [0.9,0.4],c_{1}=c_{2}=1.469$, Swarm size *Np* = 100, maxIter = 1000
A star [11]	Initial heuristic h=0 estimates the distance to the goal, Open list Ol=∅, Closed list Cl=∅
MOPSO [46]	$c_{1}=c_{2}=1.469$, ω∈[0.9,0.4], Swarm size *Np* = 100, maxIter = 1000, $V_{max}=10,V_{min}=-10$,
DWA [45]	Robot max steering velocity ϑ=0.2m/s; heading change rate ∂=0.45° s; time-to-collision ttc=0.95 s; distance to goal $d_{goal}=5.0\;m$; obstacle proximity $d_{goal}=0.8$; safe velocity $v_{safe}=0.1\;m/s$, and max acceleration $a_{max}=1.0\;m$/s

**Table 3:** Comparison of the average value results of several measures over *N* running times.

AVG (Values) Metrics	Approaches			
	A* Algorithm-DWA	NSGA-II-DWA	SPEA-DWA	MOPSO-DWA
Path length (m)	4.39 × 10^1^	4.39 × 10^1^	4.44 × 10^1^	4.30 × 10^1^
Path inflection	2	2	3	1
Collision rate	7%	9%	9%	6%
Goal-reaching rate	93%	95%	96%	97%
Smoothness of motion	42%	50%	59%	62%
Path nodes	325	325	301	260
Path planning time (s)	2.83 × 10^0^	2.83 × 10^0^	2.33 × 10^0^	2.03 × 10^0^

## Data Availability

Data are contained within the article.
